# GH Responsiveness to Combined GH-Releasing Hormone and Arginine Administration in Obese Patients with Fibromyalgia Syndrome

**DOI:** 10.1155/2017/3106041

**Published:** 2017-06-28

**Authors:** Antonello E. Rigamonti, Graziano Grugni, Marco Arreghini, Paolo Capodaglio, Alessandra De Col, Fiorenza Agosti, Alessandro Sartorio

**Affiliations:** ^1^Department of Clinical Sciences and Community Health, University of Milan, Milan, Italy; ^2^Experimental Laboratory for Auxo-Endocrinological Research, Istituto Auxologico Italiano, IRCCS, Milan and Verbania, Italy; ^3^Division of Auxology, Istituto Auxologico Italiano, IRCCS, Verbania, Italy; ^4^Orthopedic Rehabilitation Unit, IRCCS, Istituto Auxologico Italiano, IRCCS, Verbania, Italy

## Abstract

Reportedly, fibromyalgia (FM) is frequently associated with reduced IGF-1 levels and GH hyporesponsiveness to different GH stimulation tests. Since there is a high prevalence of obesity in FM, and obesity itself is characterized by hyposomatotropism, the aim of this study was to assess IGF-1 levels and GH responsiveness in sixteen severely obese women suffering from FM, who, subdivided into two subgroups on the basis of their age-dependent IGF-1 values (> or <−2 SDS), underwent the combined GHRH plus arginine test. Four out of 16 obese women with FM (25%) had low IGF-1 SDS values, 2 cases of this subgroup (12.5%) failing also to normally respond to the test. Among patients with normal GH responses, 4 showed a delayed GH peak. The subgroup with low IGF-1 SDS values had higher BMI than that with normal IGF-1 SDS. GH peak and area under the curve were not correlated with CRP, ESR, or tender point score, while significant correlations were found with fat-free mass and fat mass. In conclusion, this study shows the existence of a high prevalence of GH-IGF-1 dysfunction in patients with both FM and obesity, presumably as a consequence of the obese rather than fibromyalgic condition.

## 1. Introduction

Fibromyalgia (FM) is currently viewed as a common clinical syndrome characterized by widespread musculoskeletal pain and decreased pain threshold to pressure and other stimuli. However, the large majority of patients suffering from FM also experience other complaints, such as fatigue after mild physical efforts, morning stiffness, sleep disorders (nonrestorative sleep and insomnia), cognitive disturbances (especially, loss of memory), irritable bowel syndrome, headache, paraesthesia, and increased lifetime psychiatric comorbidity, mainly depression and anxiety disorders [1]. Depending on the diagnostic criteria used, the prevalence of FM is from 2% to 10% of the general population and about 20% among the outpatients attending rheumatology clinics [2].

Although the aetiology of FM remains unclear, low levels of insulin-like growth factor 1 (IGF-1), the main biological effector of growth hormone (GH) peripheral actions, are found in a subgroup of subjects suffering from FM [3]. Furthermore, GH stimulation tests frequently reveal a suboptimal GH responsiveness, which is however not always related with low IGF-1 levels [4].

The diagnosis of GH deficiency (GHD) is not rare in fibromyalgic patients, who often show signs and symptoms resembling the clinical picture of adult GHD [4]. As documented in some randomized placebo-controlled studies, only few months of daily recombinant human GH (rhGH) are able to reduce symptoms related to FM, including pain, to normalize IGF-1 levels and to improve quality of life [5].

Obesity is a complex disease, defined as a condition of excessive accumulation of body fat. Obesity is characterized by hyposomatotropism, as documented by reduced GH responses to physiological and pharmacological stimuli, being serum IGF-1 levels generally normal-to-high [6].

There is a strong evidence that obese individuals complain more of musculoskeletal pain and physical dysfunction than people of normal weight [7]. Obesity seems to be associated with some rheumatologic conditions, the most significant association being with FM. In this context, Ursini et al. [8] demonstrated the existence of an epidemiological correlation between these two pathological conditions, with a prevalence of obesity in fibromyalgic patients of about 40%.

This link is clinically relevant because the fibromyalgic obese group exhibits lower performance capacity and higher disability level as compared with the nonfibromyalgic obese group [9]. Obesity can be considered an aggravating comorbid condition, affecting negatively FM severity, global quality of life, fatigue, and physical dysfunction.

Weight loss induced by bariatric surgery was reported to reduce pain and tender point scores in patients with both obesity and FM, with a relevant improvement of quality of life [10]. It is noteworthy that any intervention aimed at reducing body weight in obese or overweight subjects is followed by a normalization of somatotropic function [6], which in turn might be involved in these positive effects. Current medical therapy for obesity, however, has limited effectiveness on weight loss maintenance. Consequently, some authors have proposed to use rhGH also in obesity for its lipolytic and weight-reducing effects [11]. Anyway, further studies are needed before adopting this hormone therapy in clinical practice for obese subjects.

Although the state of hyposomatotropism may be present in either obesity or FM, to our best knowledge, no study has evaluated the somatotropic function in severely obese patients suffering from FM so far. Therefore, the present study was carried out with the aim of determining the proportion of individuals with low serum IGF-1 levels and/or failing to respond to the combined GH-releasing hormone (GHRH) plus arginine test in a cohort of FM subjects with severe obesity.

## 2. Materials and Methods

### 2.1. Patients

Sixteen adult female patients, aged 18–51 years (mean ± SD: 36.5 ± 10.4 years), severely obese (body mass index, BMI range: 37.4–60.6 kg/m^2^; mean BMI: 43.6 ± 6.2 kg/m^2^), recruited at the Division of Metabolic Diseases, Istituto Auxologico Italiano, Verbania, Italy, were included in the study. All patients, who complained of FM-related symptoms since at least seven months, were diagnosed to suffer from FM by using the 2010 ACR diagnostic criteria for FM [12]. Other rheumatologic diseases were excluded by clinical examination and X-ray imaging. A part from FM and obesity, no evident signs and symptoms that can be related with clinical endocrine conditions were present. PCOS (polycystic ovary syndrome) and hypothyroidism were excluded by a pelvic echography and hormonal evaluations, as appropriate.

All patients had regular menses, and their enrolment into the study was carried out at the start of their follicular phase. No woman was taking drugs affecting GH secretion, including antidepressants, antiepileptic drugs, tramadol, and oral estrogens.

Physical examination included determination of height and weight in fasting conditions and after voiding. Standing height was determined by a Harpenden Stadiometer (Holtain Ltd., Dyfed, UK). Body weight was measured to the nearest 0.1 kg, by using standard equipment. BMI was defined as weight in kilogrammes divided by the square of height in metres. Waist circumference was measured in standing position halfway between the inferior margin of the ribs and the superior border of the crista. Hip circumference was measured as the greatest circumference around the nates.

Fat-free mass and fat mass were evaluated by bioelectrical impedance analysis (Human-IM Scan, DS-Medigroup, Milan, Italy) and expressed as kg and percentage.

The entire study protocol was approved by the ad hoc Ethical Committee of Istituto Auxologico Italiano. Written informed consent was obtained from all participants.

### 2.2. Endocrine Protocol

All subjects underwent a standard GHRH plus arginine test, which was performed within one week from the diagnosis of FM. Tests started at 08:30 AM after overnight fasting, with the patients recumbent. Fifteen minutes after an indwelling catheter had been placed in an antecubital vein, each subject received GHRH (1-29) injection (GHRH, Ferring GmbH, Kiel, Germany; 1 *μ*g/kg as i.v. bolus at 0 min). From 0 to 30 min after GHRH administration, 0.5 g/kg (maximum dose 30 g) of arginine hydrochloride (SALF, Bergamo, Italy) was infused. Blood samples for GH determination were drawn at −15, 0, 30, 45, 60, 90, and 120 min. GHD was classified according to a GH peak value to GHRH plus arginine test lower than 4.2 ng/ml [13]. This stringent criterion was used because it has been shown to have the highest values of sensitivity and specificity when diagnosing GHD in adult obesity [13] and to minimize the misclassification of obese FM patients as GH-deficient subjects when this was not the case [14].

Baseline blood samples were drawn for determination of C-reactive protein (CRP), erythrocyte sedimentation rate (ESR), and IGF-1 (see below).

IGF-1 levels were expressed as standard deviation score (SDS) from an age-matched reference value, considering those normal subjects with IGF-1 SDS above −2 and those abnormal subjects with IGF-1 SDS below −2. Depending on IGF-1 SDS values, the study population was thus divided into two subgroups (subgroup > −2 SDS: 12 subjects; subgroup < −2 SDS: 4 subjects).

### 2.3. Hormonal and Biochemical Measurements

Serum GH concentrations were determined by a commercially available immunometric chemiluminescence kit (Immulite 2000, DPC, Los Angeles, CA, USA). The international standard for recombinant human GH NIBSC Code 98/574 was used as standard material. Intra- and interassay coefficients of variation (CV) for this assay were 2.5% and 6%, respectively. The sensitivity of the method was 0.01 ng/ml.

Serum IGF-1 concentrations were determined by the commercially available immunometric chemiluminescence assay Immulite 2000 (DPC; Los Angeles, USA). The international standard for recombinant human IGF-1 NIBSC Code 02/254 was used as a standard material. Intra- and interassay CVs were 2.9% and 7.4%, respectively. The sensitivity of the assay was 20 ng/ml.

A high-sensitivity immunochemiluminescent method was used to measure serum levels of RCP (Immulite 2500, DPC; Los Angeles, USA), being the analytical sensitivity 0.01 mg/dl. The intra- and interassay CVs were <8.7% for both parameters.

ESR was measured by a microspectrophotometer with the stopped flow technique (Alifax). The intra- and interassay CVs were 5%.

For each parameter, all of a single subject's samples were run in the same assay and the order of tubes in the assay was randomized. All CVs above reported were periodically confirmed in our laboratory by using specific controls of quality.

### 2.4. Statistical Analysis

All data were presented as means (±standard deviation, SD). Areas under curve (AUCs) of GH were calculated by the trapezoidal method.

Gaussian distribution was verified for each parameter before applying any parametric statistical test. GH concentrations were compared within each group (versus basal value, that is, at T0) and among the two groups (i.e., subjects with normal or low IGF-1 SDS values) using a two-way ANOVA with repeated measures followed by the post hoc Bonferroni's test. The Student *t*-test for unpaired data was used to compare the demographic, clinical, and hormonal characteristics between the two groups with normal or low IGF-1 SDS values. Correlations between peak and AUC values of GH with the other parameters were calculated by the least squares regression approach. Statistical significance was set at *p* < 0.05.

## 3. Results

Demographic, clinical, and hormonal characteristics of the study population are reported in Table 1 (all data and subjects with normal or low IGF-1 SDS values, resp.).

Among the entire group, 3 patients had a tender point score of 13/18, 7 of 14/18, 2 of 15/18, 2 of 16/18, and 2 of 18/18.

Four out of 16 obese women with FM (25%) had low IGF-1 SDS values (PT#13, PT#14, PT#15, and PT#16). This subgroup had significantly higher BMI values than normal IGF-1 SDS values. By contrast, there were no statistically significant differences in age, fat mass, fat-free mass, hip, and waist between the two subgroups.

**Figure 1:**
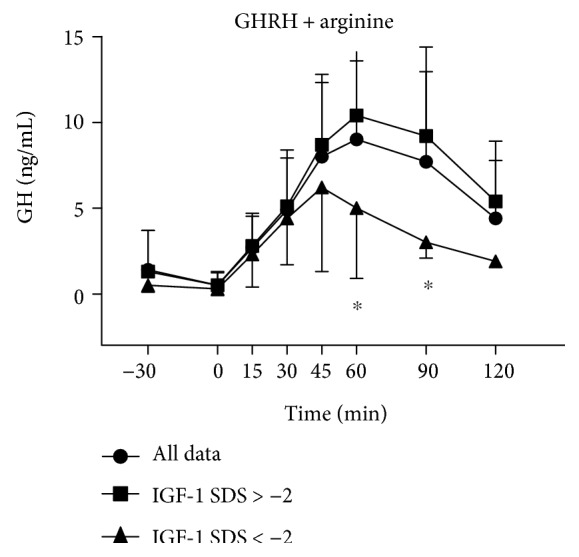
Serum concentrations of GH in FM obese patients (all data and subjects with normal or low IGF-1 SDS values), who underwent the combined GHRH plus arginine test. Data are expressed as mean ± SD. Please note that GH levels were significantly higher at T30, T45, T60, and T90 versus T0 (*p* < 0.01) when considering all data and the two subgroups with normal or low IGF-1 SDS values. ^∗^*p* < 0.05 versus the corresponding time point of the group with normal IGF-1 SDS values.

GHRH plus arginine administration evoked a significant increase in GH levels (at T30, T45, T60, and T90 versus T0, *p* < 0.05) in the entire population and in the two subgroups with normal or low IGF-1 SDS values (Figure 1). GH response in subjects with low IGF-1 SDS values was significantly reduced when compared to that in the subgroup with normal IGF-1 SDS values (at T60 and T90, *p* < 0.05) (Figure 1).

**Figure 2:**
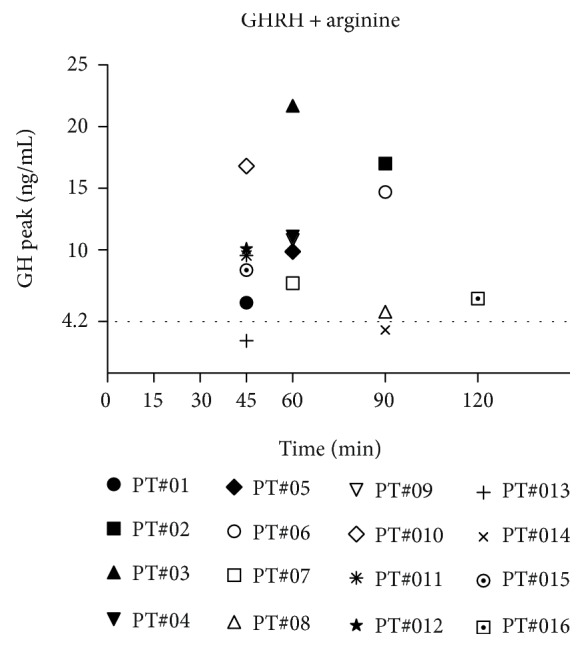
GH peaks obtained in each of the 16 FM obese patients who underwent the GHRH plus arginine test together with the corresponding time points (i.e., *T*_max_).

Considering all patients, the rate of failure in GH response to the combined GHRH plus arginine test was 12.5% (2 out of 16 patients, PT#13 and PT#14, both belonging to the subgroup with low IGF-1 SDS values) (Figure 2). Among patients with normal GH responses, 4 showed a delayed GH peak (3 at T90 and 1 at T120) (Figure 2). A delayed GH peak was also found in 1 subject with GHD (at T90) (Figure 2).

The two GH deficient patients were further investigated to exclude the existence of other pituitary hormonal deficiencies. No other pituitary deficiencies were found.

The most relevant significant correlations were the ones between peak GH or GH AUC and height (*r* = 0.656 and *r* = 0.657, *p* < 0.01), fat-free mass in kg (*r* = 0.564 and *r* = 0.555, *p* < 0.01), fat-free mass in percent (*r* = 0.492 and *r* = 0.554, *p* < 0.05), and fat mass in percent (*r* = −0.492 and *r* = −0.554, *p* < 0.05), respectively. On the contrary, no correlations between GH peak or GH AUC and tender point score, ESR, and CRP were found. Finally, peak GH was positively correlated with IGF-1 levels (*r* = 0.133, *p* < 0.05).

## 4. Discussion

A significant number of fibromyalgic patients are reported to have a somatotropic dysfunction [4]. Particularly, Cuatrecasas et al. [3] found reduced IGF-1 levels (150 ng/ml or less) in approximately 34% of overweight (BMI = 27.2 ± 4.1 kg/m^2^) patients with severe FM. The same authors reported a reduced GH peak (< 5 ng/ml) to insulin-induced hypoglycaemia test in 17% of subjects, 6% of patients having a GH peak equal to or less than 3 ng/ml [3]. In another clinical study performed in fibromyalgic patients with a mean BMI of about 30 kg/m^2^, 17% of patients tested with GHRH plus arginine failed to respond above a cutoff of 4.2 ng/ml [14].

Differently from the above reported cohorts, the present study was carried out in a group of patients with both severe obesity (mean BMI: 43.6 ± 6.2 kg/m^2^) and FM. Twenty-five percent of our subjects had markedly low IGF-1 levels (less than −2 SDS, according to age), while 12.5% of them were GH deficient, accordingly with a cutoff value of 4.2 ng/ml, which is suggested to be used when testing obese subjects [13]. Moreover, a delayed GH peak was found in 5 patients (31%), 4 with normal GH-stimulated levels, and 1 showing an impaired GH response to the stimulus. This delayed GH peak is an important finding because it can be considered suggestive of a dysfunction in the hypothalamic regulation network of GH secretion, involving the interplay of both GHRH and somatostatin tone [15].

Our results (i.e., the prevalence of low IGF-1 levels and the rate of GHD in patients with both FM and obesity) are slightly lower than or similar to those obtained in fibromyalgic patients with lower BMI (i.e., lean, overweight, and moderately obese subjects), thus suggesting that the there is no “additive” effect between the hyposomatotropism present in obesity and the somatotropic dysfunction in FM. These data are also in line with those reported by Maccario et al. [16], who showed a reduction of GH response to GHRH plus arginine in 33.9% of (nonfibromyalgic) obese subjects (taking into account the 1st centile limit of normal response) and low IGF-1 levels in 22%. In the same work, considering 3.0 ng/ml as arbitrary cutoff, GH response was reduced in 5.7% of obese patients, one of them having low IGF-1 levels (1.9%).

Based on these considerations, we may argue that the mechanisms underlying the hyposomatotropism in obesity and the somatotropic dysfunction in FM are likely to be common, presumably an imbalance in neurotransmitters and/or neuropeptides at the sovra- or intrahypothalamic level, an alteration which has been invoked to explain some symptoms present in FM, such as chronic pain, sleep disturbances, psychiatric disorders (mainly anxiety and depression), and other neuroendocrine dysfunctions [17].

In this light, it is interesting to recall that pyridostigmine, an anticholinesterase inhibitor, capable of reducing hypothalamic somatostatin tone, was reported to improve exercise-induced GH release in patients suffering from FM, thus suggesting the existence of a somatostatin hypertone, a condition that has been supposed to occur also in obesity [18].

This interpretation is, obviously, valid for the cases of GHD deriving from neurosecretory dysfunction and not organic hypothalamus-pituitary diseases, which can be identified in fibromyalgic patients with GHD [14].

A high prevalence of obesity has been found in FM, and obesity is considered to be a relevant risk factor for FM [8]. In this context, it is noteworthy that the majority of GH-deficient cases reported in the literature have often have a BMI > 30 kg/m^2^ [3, 14]. Furthermore, in the present study, the group with low IGF-1 SDS values had significantly higher BMI values when compared to the group with normal IGF-1 levels. Therefore, it is difficult to refer the high prevalence of impairment in GH-IGF-1 axis to FM itself or to obesity that is often associated with this rheumatologic condition. In our study, the correlations of GH peak with some parameters of body composition, together with the lack of any correlations between GH peak and clinical (tender point score) or biochemical (ESR and CRP) indices of FM severity, seem to suggest that the latter hypothesis (i.e., obesity causing the most cases of GHD or impairment in GH-IGF-1 axis) is more convincing than the former one (i.e., FM causing the most cases of GHD or impairment in GH-IGF-1 axis). However, the association of both low IGF-1 SDS values and absent/low GH-stimulated response in the same individuals is suggestive of a clear-cut, though functional, GH deficient status. In this respect, it is noteworthy that low IGF-1 levels and altered GH-stimulated secretion in obesity seem to identify a subset of subjects with an increased cardiovascular risk [6].

Yuen et al. [14] showed that majority of FM patients with low IGF-1 have an exuberant GH response to the GHRH plus arginine stimulation, implying a diagnosis of GH resistance. In our study, among the group with low IGF-1 levels, only one patient (PT#16) exhibited a GH peak GH > 10 ng/ml (at T45). Therefore, using the GHRH plus arginine test might have overestimated GH response in any patient, given the potential hypothalamic effects of arginine on the “dysregulated” GH/IGF-1 axis present in FM. Further studies with the adoption of different GH provocative tests are mandatory to solve this intriguing issue.

As far as our study is concerned, some limitations are to be considered. Firstly, we have recruited a limited number of patients; therefore, our results should be considered only preliminary. Secondly, we have enrolled only female patients; since GH secretion is differently regulated by sex steroid hormones [15], the present study should be replicated in a group of male subjects, though the low prevalence of FM in men might hamper the selection of an adequate number of male subjects with both severe obesity and FM [1]. The third issue is that, in the present study, to assess the body composition, we used bioelectrical impedance analysis throughout population-specific equations developed in our laboratory [19]. This method usually presents some limitations in morbidly obese patients, due to the relatively increased amount of total body water and a relative increase in extracellular water, which can result in an underestimation of the percentage of body fat and an overestimation of fat-free mass in these subjects. Last of all, data about hypothalamic-pituitary morphology are lacking due to technical problems related to the extremely high BMI values of the GHD patients, thus hampering to define the aetiology of the impaired GH secretion in a better way.

Differently from other clinical studies on this topic recruiting patients taking antidepressants, antiepileptic drugs, tramadol, estrogens, and other medications, which could have introduced biases for the relevant effects of these drugs on GH secretion [4], a positive aspect of the present study is represented by the fact that our patients were taking no medications known to affect the endocrine system, including the somatotropic axis.

In conclusion, the present study shows that a not negligible subpopulation (25%) of patients with both FM and severe obesity has low IGF-1 SDS values, 12.5% failing also to normally respond to the GHRH plus arginine test. This seems to be a consequence of the obese rather than fibromyalgic condition, a conclusion that should be cautiously considered because of the small number of patients recruited. Although the results of the present study are preliminary, GH responsiveness of patients with both FM and severe obesity should be carefully evaluated, since GH replacement therapy might potentially improve the clinical symptoms and the general well-being of those with GHD [5, 20].

## Figures and Tables

**Table 1:** Demographic, clinical, and hormonal characteristics of the obese women with fibromyalgia syndrome included in the study.

Parameter	All data	IGF-1 SDS > −2	IGF-1 SDS < −2
Number	16	12	4
Age (yrs)	36.5 ± 10.4	36.5 ± 10.5	36.8 ± 11.8
BMI (kg/m^2^)	43.6 ± 6.2	41.8 ± 4.2	48.9 ± 8.8^∗^
Waist (cm)	126.4 ± 11.1	123.9 ± 9.9	134.0 ± 12.6
Hip (cm)	133.8 ± 13.1	131.1 ± 6.4	141.8 ± 24.5
Fat mass (kg)	61.6 ± 12.9	58.6 ± 8.2	70.6 ± 19.8
Fat mass (%)	54.1 ± 4.7	53.0 ± 4.4	57.2 ± 4.6
Fat-free mass (kg)	51.6 ± 4.2	51.5 ± 3.6	51.7 ± 6.3
Fat-free nass (%)	46.0 ± 4.7	47.0 ± 4.4	42.8 ± 4.6
Tender-point score	14.7 ± 1.6	14.5 ± 1.5	15.3 ± 1.9
ESR (mm/h)	33.9 ± 18.7	34.8 ± 19.5	31.5 ± 18.7
CRP (mg/dl)	1.2 ± 1.2	0.9 ± 0.8	2.1 ± 2.1
IGF-1 (ng/ml)	138.1 ± 71.0	160.7 ± 66.2	70.5 ± 31.2^∗^
IGF-1 SDS	−1.1 ± 0.9	−0.7 ± 0.7	−2.3 ± 0.1^∗^
Peak GH (ng/ml)	10.4 ± 5.2	11.7 ± 5.0	6.7 ± 4.5
GH AUC (ng × min/ml)	716.7 ± 392.4	812.8 ± 391.4	428.3 ± 245.6^∗^

^∗^ *p* < 0.05 versus group with IGF-1 SDS > −2. BMI: body mass index; ESR: erythrocyte sedimentation rate; CRP: C-reactive protein; IGF-1: insulin-like growth factor 1; GH: growth hormone; AUC: area under the curve.

## Additional Points

*Availability of Data and Materials*. All data from this study is included in the figures and the table of the manuscript.
